# Arabidopsis Protein Phosphatase DBP1 Nucleates a Protein Network with a Role in Regulating Plant Defense

**DOI:** 10.1371/journal.pone.0090734

**Published:** 2014-03-04

**Authors:** José Luis Carrasco, María José Castelló, Kai Naumann, Ines Lassowskat, Marisa Navarrete-Gómez, Dierk Scheel, Pablo Vera

**Affiliations:** 1 Instituto de Biología Molecular y Celular de Plantas, Universidad Politécnica de Valencia-C.S.I.C, Ciudad Politécnica de la Innovación, Valencia, Spain; 2 Department of Stress and Developmental Biology, Leibniz Institute of Plant Biochemistry, Halle, Germany; Iwate University, Japan

## Abstract

*Arabidopsis thaliana* DBP1 belongs to the plant-specific family of DNA-binding protein phosphatases. Although recently identified as a novel host factor mediating susceptibility to potyvirus, little is known about DBP1 targets and partners and the molecular mechanisms underlying its function. Analyzing changes in the phosphoproteome of a loss-of-function *dbp1* mutant enabled the identification of 14-3-3λ isoform (GRF6), a previously reported DBP1 interactor, and MAP kinase (MAPK) MPK11 as components of a small protein network nucleated by DBP1, in which GRF6 stability is modulated by MPK11 through phosphorylation, while DBP1 in turn negatively regulates MPK11 activity. Interestingly, *grf6* and *mpk11* loss-of-function mutants showed altered response to infection by the potyvirus *Plum pox virus* (PPV), and the described molecular mechanism controlling GRF6 stability was recapitulated upon PPV infection. These results not only contribute to a better knowledge of the biology of DBP factors, but also of MAPK signalling in plants, with the identification of GRF6 as a likely MPK11 substrate and of DBP1 as a protein phosphatase regulating MPK11 activity, and unveils the implication of this protein module in the response to PPV infection in Arabidopsis.

## Introduction

DNA-binding protein phosphatases (DBPs) are a unique family of protein phosphatases of the 2C class that are distinctively capable of binding DNA [1]. While protein phosphatase activity lies in the C-terminal domain, the ability to bind DNA resides in an N-terminal extension, which is barely conserved beyond a motif directly involved in binding [1], [2]. DBP factors, although apparently unique to plants, are present throughout the plant kingdom and seem to have functionally diversified during evolution [2].

Tobacco DBP1 (NtDBP1), the founding member of this family, was shown to be involved in the transcriptional regulation of a defense-related gene in the course of compatible plant-virus interactions [1]. More recently, in *Arabidopsis thaliana*, DBP1 was found to mediate disease susceptibility to potyviruses like *Plum pox virus* (PPV) and *Turnip mosaic virus* (TuMV) [3]. Moreover, DIP2, a small peptide of unknown function that functionally modulates DBP1 in Arabidopsis, was identified as an additional host factor influencing PPV infection, thereby emphasizing the significant implication of DBP1 in the interaction [4]. DBP1 function is also modulated through the interaction with 14-3-3 proteins, highly conserved ubiquitous eukaryotic proteins recognized as important mediators in the regulation of diverse biological processes, in particular in signal transduction and transcription [5]. Carrasco et al (2006) reported that both in tobacco and Arabidopsis, DBP1 orthologs directly interact with 14-3-3G and 14-3-3λ isoforms, respectively, and this interaction provokes the nuclear export of the complex to the cytoplasm [6]. Retention of DBP1 in the cytosol will in turn prevent binding of DBP1 to the promoter region of defense-related target genes, eventually relieving gene repression and concomitantly promoting function of DBP1 in the cytosol.

Mitogen-activated protein kinase (MAPK) cascades are essential components of signal transduction mechanisms evolutionarily conserved in all eukaryotes. A typical MAPK module consists of three protein kinases, MAP kinase kinase kinase (MAP3K), MAP kinase kinase (MAPKK) and MAPK, which sequentially phosphorylate and activate each other to mediate cell responses and developmental pathways [7]. Once activated, MAPK phosphorylates and modulates specific targets, such as transcription factors. MAPK activation occurs through conserved threonine and tyrosine phosphorylation by MAPKK, a dual specificity kinase [8]. Dephosphorylation of either residue by specific protein phosphatases inactivates MAPK [9].

The present study provides further insights into the molecular mechanisms underlying DBP1 function by comparing the phosphoproteome of loss-of-function *dbp1* mutant plants to wild type. Among the detected proteins, we identified 14-3-3λ (GRF6), an earlier described DBP1 interactor, and MPK11, a MAPK activated during the plant immune response [10]. Physical interactions among these proteins were demonstrated *in vivo* by Bimolecular Fluorescence Complementation analyses and coimmunoprecipitation. We showed that DBP1 negatively regulated MPK11 activity, and that MPK11 mediated phosphorylation of GRF6, promoting ubiquitination and increased GRF6 protein turn-over. These results unveil a phosphorylation-dependent, proteasome-mediated regulatory mechanism of GRF6, and identify a likely substrate for MPK11, a MAPK with unknown function, whose activity is shown to be, in turn, modulated by DBP1. Interestingly, reverse genetic studies revealed that both GRF6 and MPK11 are involved in the response of Arabidopsis plants to infection by the potyvirus *Plum pox virus*, and the described regulation of GRF6 protein stability by MPK11 was shown to occur during PPV infection, appearing as a novel signalling component in the Arabidopsis-PPV interaction.

## Materials and Methods

### Plant Material and Growth Conditions

Arabidopsis plants were grown in a growth chamber under a 10/14 h light/dark photoperiod (23 to 19°C, 85% relative humidity, 100 µE m^−2^ s^−1^ fluorescent illumination). All mutants were in a Col-0 background and were obtained from the European Arabidopsis Stock Center at the University of Notthingham (http://arabidopsis.info/). *dbp1* mutant was already described [3]. *mpk11* and *grf6* correspond to SALK_049352 and SALK_075219 lines, respectively (Salk Institute Genomic Analysis Laboratory, http://signal.salk.edu/).

### Phosphoprotein Enrichment and Analysis

A detailed description of the protocol used for phosphoprotein enrichment is included in Supporting Information.

### Transient Expression in *Nicotiana benthamiana* Leaves

Tobacco plants (*Nicotiana benthamiana*) were grown in a phytochamber under short day conditions at 23°C/19°C. Almost fully expanded leaves were infiltrated with a suspension of *Agrobacterium tumefaciens* C58 bearing the relevant construct in 10 mM MES pH 5.6, 10 mM MgCl_2_, 150 µM acetosyringone at an OD_600_ = 0.5. After 3 days, fluorescence was analyzed in infiltrated leaves by confocal microscopy. For co-infiltration, *Agrobacterium* cultures grown separately and processed as indicated above, were adjusted to an O.D. = 0.5, and mixed prior to infiltration. *Agrobacterium* expressing the viral silencing suppressor P19 was included in all infiltrations.

### Fluorescence Microscopy

GFP/YFP fluorescence in inoculated plants was monitored using Nikon SMZ800, and Leica MZ16F microscopes.

### Bimolecular Fluorescence Complementation Analysis

The relevant gene coding sequences were amplified by PCR using specific oligonucleotides bearing *attB* sites, cloned into pDONR207 (Invitrogen), and finally transferred to pYFN43 and pYFC43 plasmids created by A. Ferrando (IBMCP, Valencia, Spain; http://www.ibmcp.upv.es), to generate C-terminal translational fusions to the N-terminal (YFP^N^) and C-terminal (YFP^C^) fragments of the yellow fluorescent protein (YFP), respectively. Once transferred to *Agrobacterium*, constructs were transiently expressed in *N. benthamiana* leaves as described above. Fluorescence was detected by confocal microscopy three days following infiltration.

### Viral Inoculation

PPV was inoculated by gently making a single puncture on the distal part of a leaf with a sterile toothpick soaked in a suspension of *A. tumefaciens* bearing an infectious cDNA clone at an O.D. = 1 at 600 nm. The *Agrobacterium* suspension was prepared as described above.

### RNA Extraction, RT and qPCR

Total RNA was extracted using TRIzol® reagent (Invitrogen) following the manufacturer’s recommendations and further purified by lithium chloride precipitation. For reverse transcription, the RevertAid™ H Minus First Strand cDNA Synthesis Kit (Fermentas Life Sciences) was used. Quantitative PCR (qPCR) amplifications and measurements were performed using an ABI PRISM 7000 sequence detection system, and SYBR-Green (Perkin-Elmer Applied Biosystems). *ACTIN2/8* was chosen as the reference gene. The following oligonucleotides were utilized in the analyses:

PPV-CP-FW: 5′-GACTACGGCGTCAATGCTCAAC-3′

PPV-CP-RV: 5′- GTTTGCAGTTGAGGTCCTGACAC-3′

ACTIN2/8-FW: 5′-GGTAACATTGTGCTCAGTGGTGG-3′

ACTIN2/8-RV: 5′-AACGACCTTATCTTCATGCTGC-3′

MPK4-FW: 5′-TTCCCAAACATGTCGGCTGGTG-3′

MPK4-RV: 5′-TGGCACAACGCCTCATCAACTG-3′

MPK11-FW: 5′-ACCCAAACAGACGCATTACAGTCG-3′

MPK11-RV: 5′-TCGTGTAGCGGTGCTAAGTAAGG-3′

GRF6-FW: 5′-TATGCAGGAGCAGATGGACGAG-3′

GRF6-RV: 5′-GGTGGCAGAAACATCGCGTAAC-3′

### Western Blot and Immunoprecipitation

Protein crude extracts were prepared by homogenizing ground frozen leaf material with Tris-buffered saline (TBS) supplemented with 5 mM DTT, protease inhibitor cocktail (Sigma-Aldrich), and protein phosphatase inhibitors (PhosStop, Roche). Protein concentration was measured using Bradford reagent; 25 µg of total protein was separated by SDS-PAGE (12% acrylamide w/v) and transferred to nitrocellulose filters. The filter was stained with Ponceau-S after transfer, and used as a loading control.

For immunoprecipitations, extracts were cleared by two successive centrifugation steps at 16000×g and 4°C for 30 minutes, and then incubated at 4°C for 2 h with conjugated antibodies under gentle rocking. Unbound proteins were removed by successively washing the resin with TBS, TBS 0.5% (v/v) Triton X-100 and TBS 0.3M NaCl, 1% (v/v) NP-40. We used monoclonal anti-HA clone HA-7 agarose-conjugate (Sigma-Aldrich), and rabbit polyclonal GFP-sepharose conjugate (Abcam). Immunoprecipitated proteins were eluted with Laemmli sample buffer by heating for 5 minutes at 95°C. For Western blots analysis the following antibodies were used: mouse monoclonal anti-GFP antibodies (Roche), rabbit polyclonal anti-MaBP antibodies (New England Biolabs), mouse monoclonal anti-phosphoMyBP clone P12 antibodies (Millipore), rabbit polyclonal anti-HA antibodies (Sigma), mouse monoclonal anti-phosphoserine Q5 antibodies (Qiagen), and mouse monoclonal anti-ubiquitin Ub (P4D1) antibodies (Santa Cruz Biotechnology).

### MAP Kinase Activity Assay

The assay was performed after MPK11 immunoprecipitation, and prior to elution. Resin was resuspended with a reaction mix consisting of 50 mM Tris-HCl pH 7.5, 10 mM MgCl_2_, 1 mM DTT, 0.1 mM ATP and 0.4 mg/ml myelin basic protein (MyBP). Reactions were incubated for 30 min at 30°C in a shaking incubator. Following incubation Laemmli sample buffer was added, and samples were heated for 5 minutes at 95°C. Phosphorylated MyBP was detected by Western blot using a specific mouse monoclonal antibody as described above, following manufacturer’s instructions.

## Results

### *dbp1* Mutant Phosphoproteome Analysis

DBP1 is a protein phosphatase 2C of the DNA-binding DBP family. Changes in the phosphoproteome of *DBP1*-defective plants could reveal direct or indirect targets of DBP1. Consequently, the phosphoproteome of wild-type Col-0 and *dbp1* mutant plants was analysed applying an optimized workflow for highly efficient phosphoprotein enrichment from adult plant leaves, including ammonium sulfate precipitation and metal-oxide affinity chromatography [11] (see Methods S1). For a sensitive and detailed shotgun analysis, LTQ Orbitrap Velos technology was used on three biological replicates per genotype and relative quantification based on spectral count was performed. Efficiency and reproducibility of this procedure was analyzed by 1-D-SDS-PAGE and subsequent ProQ Diamond and CBB staining. As listed in Table SI, 169 (phospho)proteins showed differential accumulation in the *dbp1* mutant relative to Col-0 (t-test, p<0.05). Interestingly, among the identified proteins an earlier described DBP1 interactor was found. 14-3-3λ (GRF6; At5g10450) was previously shown to specifically interact with DBP1 *in vivo* [6]. In addition, identification of the MAP kinase pair MPK4 (At4g01370)/MPK11 (At1g01560) was also particularly significant since it provides an insight into the signalling processes DBP1 might be part of. However, protein identification in this case is ambiguous due to the high sequence similarity between MPK4 and MPK11. Consistent with an 89% identity between these two proteins, most of the tryptic peptides identified were present in both. Therefore, at this stage we could not ascertain the actual relationship of these two MAPKs with DBP1. MAPKs are key components of signal transduction pathways in eukaryotes. MPK4 is activated in response to pathogen-associated molecular patterns (PAMPs) and has been reported to be involved in plant immunity as a negative regulator of the salicylic acid (SA)-mediated defense pathway [12], [13]. MPK11 was also found to be activated following application of the PAMP molecule flg22 [10], but the signalling pathways in which it operates remain unknown, as unidentified are its protein targets. As previously reported, the *mpk4* mutant showed severe dwarfism (Figure S1B) [12], [14]. Therefore, since DBP1 has not been related so far to any developmental alteration and the *dbp1* mutant exhibits wild-type morphology and architecture, we focused on the *mpk11* mutant for further studies.

### Subcellular Localization and Analysis of Protein-protein Interactions

**Figure 1:**
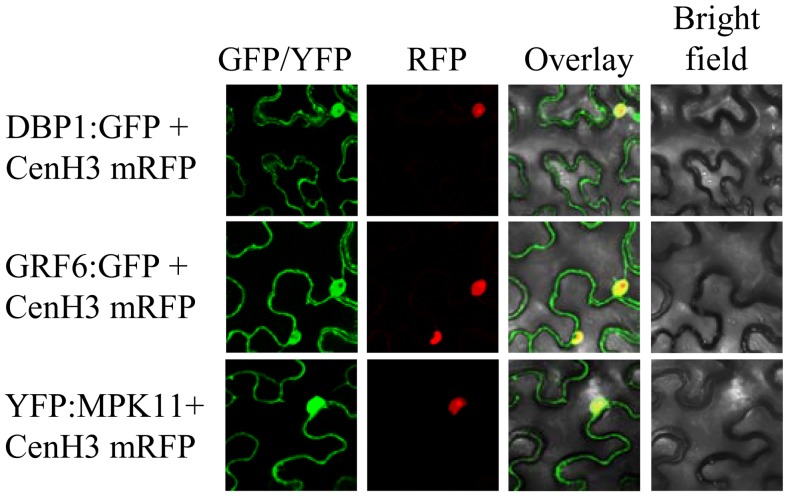
Subcellular distribution of the identified DBP1 putative targets. Full-length coding sequences were fused to the N-terminus of green fluorescent protein (GFP) or to the C-terminus of yellow fluorescent protein (YFP), and transiently expressed in *Nicotiana benthamiana* leaves by agroinfiltration. Leaf sections were inspected by confocal microscopy 3 d after infiltration. Fusion proteins were expressed in epidermal cells. A large central vacuole occupies most of the cellular space, constraining cytosol to a narrow peripheral area. Co-expression with mRFP-tagged centromeric histone H3 reveals nuclei localization.

**Figure 2:**
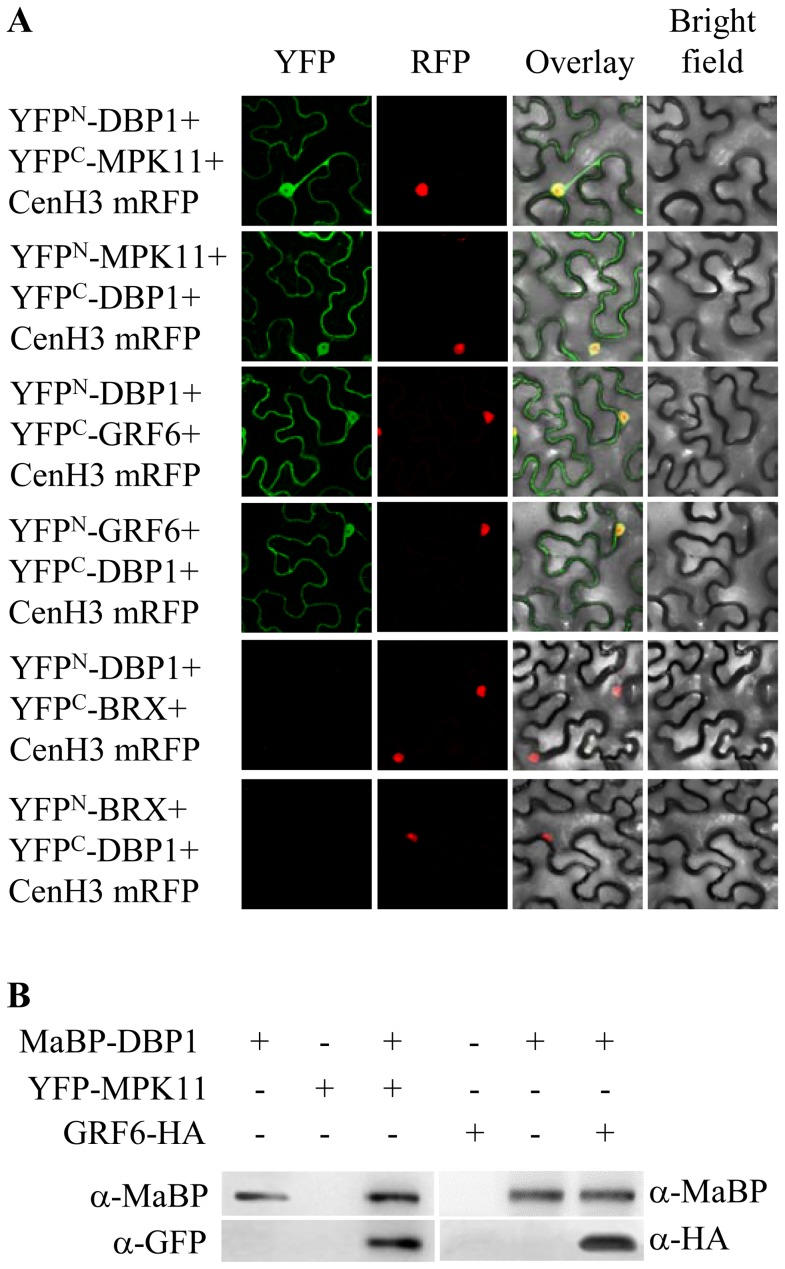
Identified proteins interact *in vivo* with DBP1. A, BiFC assay with YFP fusions transiently expressed in *N. benthamiana* leaves by agroinfiltration. Both reciprocal combinations are shown for each interaction. As a negative control, DBP1 and the unrelated transcription factor BREVIS RADIX (BRX) YFP fusions were used. Centromeric histone H3 fused to mRFP was co-expressed as a marker for nuclear localization. B, Coimmunoprecipitation validated the reported interactions. Relevant fusion proteins were transiently expressed in *N. benthamiana* leaves by agroinfiltration as indicated, and immunoprecipitated with anti-MaBP antibodies. Western blots of the immunoprecipitated fractions decorated with the indicated antibodies are shown. Anti-GFP antibodies allow for detection of YFP.

The differential protein accumulation observed in the *dbp1* mutant might reveal a direct or indirect effect of the absence of DBP1 protein phosphatase activity. Therefore, we analysed whether the selected proteins interact with DBP1 *in vivo* using Bimolecular Fluorescence Complementation (BiFC). The relevant proteins must co-localize to interact, and localization might be altered as a consequence of the interaction. Therefore, we first examined the subcellular localization of MPK11 and GRF6 when transiently expressed as GFP fusions in *Nicotiana benthamiana* leaves by agroinfiltration as described [4]. Both tobacco and Arabidopsis DBP1 localize in the nucleus and the cytosol [6]. Here, the selected proteins exhibited a similar subcellular distribution (Fig. 1). Nuclear localization was verified using centromeric histone H3 fused to Red Fluorescent Protein (mRFP) as a nuclear marker [15]. Therefore, the respective localization patterns enabled interaction with DBP1. BiFC suggested that the interactions actually occur *in vivo* after transiently expressing the relevant proteins fused to the N-terminal and C-terminal fragments of the Yellow Fluorescent Protein (YFP) in *N. benthamiana* by agroinfiltration. The interaction results in YFP fluorophore reconstitution and a fluorescence signal where the protein complex accumulates. Fig. 2A depicts confocal microscopy images obtained following coexpression of the indicated fusion proteins. DBP1-GRF6 and DBP1-MPK11 complexes were detected both in the cytosol and in the nucleus. As above, mRFP-tagged centromeric histone H3 was included in the analysis as indicative of nuclear localization. For GRF6, this was consistent with previous observations that tobacco GRF6 and DBP1 homologues interacted to mediate nucleocytoplasmic shuttling and DBP1 accumulation in the cytosol [6]. Interactions were validated by coimmunoprecipitation of fusion proteins transiently expressed in leaves of *N. benthamiana* (Fig. 2B and Figure S2)). DBP1 fused to maltose binding protein (MaBP) was immunoprecipitated with anti-MaBP antibodies. When coexpressed with MPK11 fused to YFP, or GRF6 tagged with the hemagglutinin (HA) epitope, presence of the putative partner in the immunoprecipitate was demonstrated by Western blot using anti-GFP and anti-HA antibodies, respectively. Therefore, both MPK11 and GRF6 interact *in vivo* with DBP1.

### DBP1 Inactivates MPK11 *in vivo*

**Figure 3:**
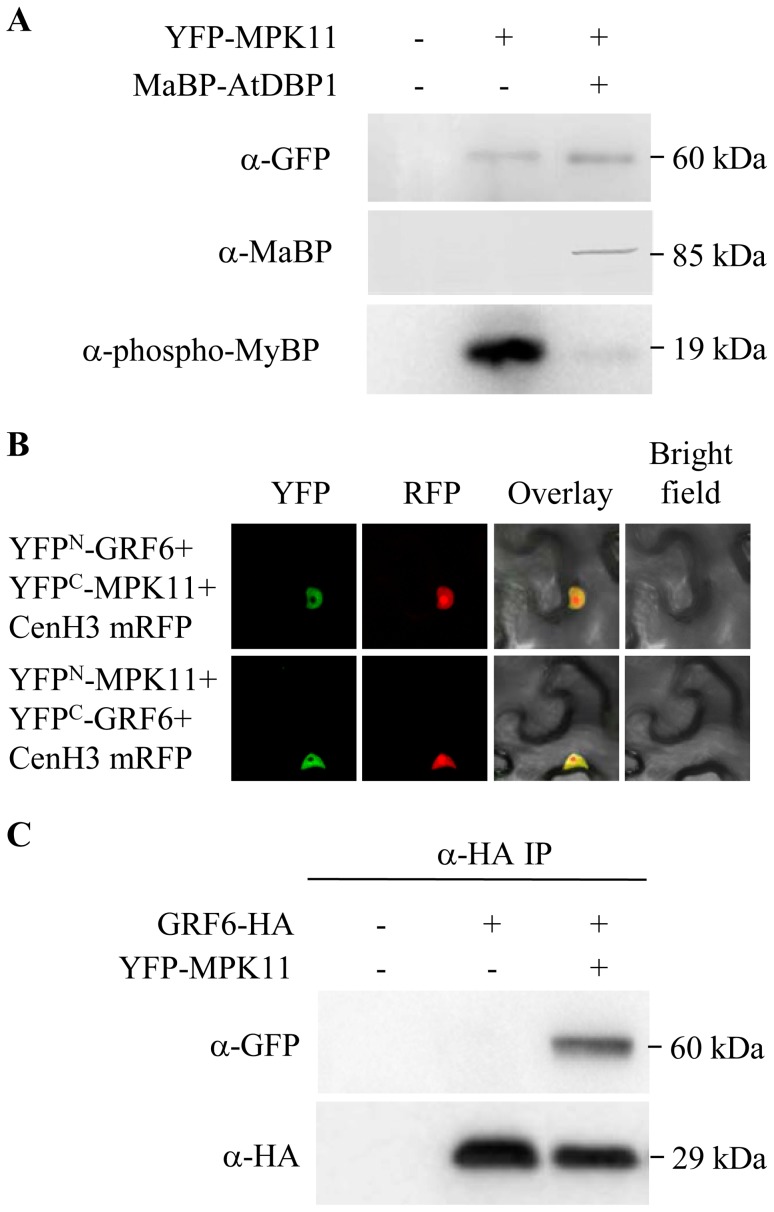
MPK11 interacts with GRF6 and is negatively modulated by DBP1. A, DBP1 inhibited MPK11 kinase activity. MPK11 and DBP1 were transiently expressed as translational fusions to YFP and MaBP, respectively, in *N. benthamiana* leaves by agroinfiltration. YFP-MPK11 was immunoprecipitated 3 d after infiltration with anti-GFP antibodies and MAPK activity was measured in the immunoprecipitate by Western blot with antibodies specifically recognizing phosphorylated MyBP (lower panel). A fraction of the immunoprecipitated protein was analyzed using anti-GFP (upper panel) and anti-MaBP (middle panel) antibodies. B, MPK11 interacted with GRF6 *in vivo*. BiFC analysis in *N. benthamiana* leaves showing specific MPK11-GRF6 interaction. The MPK11-GRF6 complex was detected predominantly in the nucleus. C, GRF6 and MPK11 co-immunoprecipitation. GRF6-HA was expressed in *N. benthamiana* leaves and immunoprecipitated with anti-HA antibodies. YFP-MPK11 was detected by Western blot with anti-GFP antibodies in the anti-HA immunoprecipitate when coexpressed with GRF6 (upper panel), confirming interaction between the two proteins. Lower panel shows GRF6-HA immunodetection in the immunoprecipitate by Western blot.

Since MAPK activity is regulated by phosphorylation and DBP1 is a protein phosphatase, DBP1 might inactivate MPK11. To test this hypothesis, we transiently expressed MPK11 in *N. benthamiana* as a YFP N-terminal fusion, alone or in combination with DBP1 fused to MaBP. MPK11 was then immunoprecipitated with anti-GFP antibodies, and MAPK activity was measured in the immunoprecipitate using myelin basic protein (MyBP) as a substrate and anti-phospho-MyBP antibodies for product detection. The experiment was repeated three times. Albeit the level of activity of MPK11 was variable, likely because activation of expressed MPK11 relies on endogenous kinases from *N. benthamiana*, the detected activity was markedly reduced in every case when DBP1 was coexpressed with MPK11. As shown in Fig. 3A for a representative experiment, similar MPK11 protein amounts were immunoprecipitated in both cases (upper panel), but when coexpressed with DBP1 (middle panel), MPK11 activity was significantly reduced (lower panel). Therefore, DBP1 is a protein phosphatase directly regulating MPK11 activity.

Since DBP1 inhibited MPK11, differential (phospho)protein accumulation in Col-0 and the *dbp1* mutant might not exclusively reflect direct DBP1-mediated dephosphorylation, but also indirect effects of increased MPK11 activity in the mutant. For that reason, and to help clarify how this emerging protein network operates, we analysed if GRF6 might be a MPK11 substrate. Using BiFC, we first showed that GRF6 interacted with MPK11 *in vivo* (Fig. 3B). The complex largely accumulated in the nucleus, indicating that the interaction likely occurred in the nuclear compartment. The interaction was confirmed by coimmunoprecipitation (Fig. 3C). When coexpressed by agroinfiltration in *N. benthamiana* together with GRF6-HA, YFP-MPK11 was detected by Western-blot in the anti-HA immunoprecipitate.

### MPK11 Phosphorylates GRF6 *in vivo*

**Figure 4:**
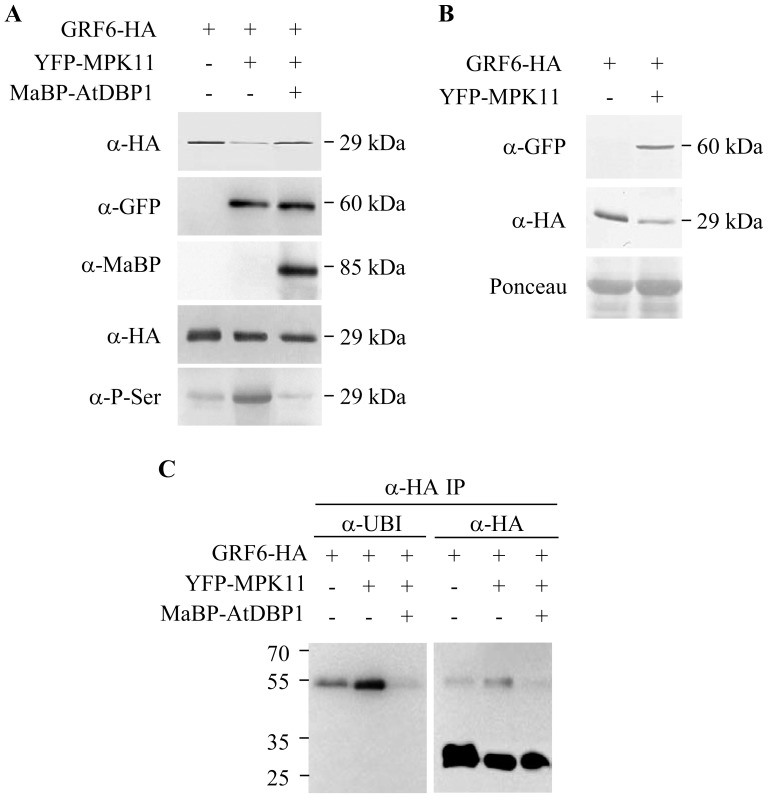
MPK11 phosphorylated GRF6 promoting ubiquitination and increased turnover. A, Detection of GRF6 phosphorylation. GRF6-HA was transiently expressed, alone or together with YFP-MPK11 and MaBP-DBP1 as indicated in *N. benthamiana* leaves by agroinfiltration, and immunoprecipitated using anti-HA antibodies. The three upper panels show detection by Western blot of the different expressed proteins with the indicated antibodies. The two lower panels show immunoprecipitation of GRF6-HA using anti-HA antibodies: an aliquot of the immunoprecipitated fraction was loaded above as a loading control, whereas in the bottom panel serine phosphorylation detected using anti-phosphoserine monoclonal antibody is shown. B, MPK11 mediated increased GRF6 turnover. GRF6-HA and YFP-MPK11 were expressed in *N. benthamiana* leaves by agroinfiltration. Western blot of protein extracts from infiltrated leaves 3 d post-infiltration immunodecorated with the indicated antibodies for specific expressed protein detection. Ponceau-S staining of the nitrocellulose membrane is shown at the bottom. C, GRF6 was ubiquitinated *in vivo* and MPK11 increased ubiquitination rate. The indicated proteins were transiently expressed in *N. benthamiana* leaves by agroinfiltration, and GRF6-HA was immunoprecipitated with anti-HA antibodies 3 d post-infiltration. *Left panel*, Western blot of the anti-HA immunoprecipitate immunodecorated with anti-ubiquitin antibodies. *Right panel*, the same blot shown in the left panel was stripped, and immunodecorated with anti-HA antibodies, indicating that the detected ubiquitinated protein corresponds to GRF6-HA and revealing unmodified GRF6.

The MPK11-GRF6 interaction suggested that GRF6 might be a MPK11 substrate. To verify this hypothesis, GRF6 phosphorylation was analyzed by Western blot after immunoprecipitation, using two different monoclonal antibodies recognizing phosphorylated serine and threonine residues. The anti-phosphoserine and anti-phosphothreonine antibodies are commercial antibodies which recognize the respective phosphorylated residues regardless of surrounding amino acids. Differences were only found in serine phosphorylation. GRF6 was phosphorylated at serine residues when expressed in *N. benthamiana* by an endogenous kinase activity (Fig. 4A, left lane, bottom panel). Serine phosphorylation significantly increased when GRF6was coexpressed with YFP-MPK11 (Fig. 4A, middle lane), suggesting additional phosphorylation as mediated by MPK11. Whether phosphorylation may be occurring directly or indirectly via another kinase activity that is modified by MPK11 remains still unknown. In the presence of DBP1, the MPK11-dependent increase in GRF6 serine phosphorylation was prevented (Fig. 4A, right lane), further supporting MPK11 inactivation by DBP1.

### MPK11 Promotes Degradation of GRF6 *in vivo*

When transiently coexpressed with MPK11 in *N. benthamiana*, GRF6 reproducibly accumulated at lower levels than when expressed alone (Fig. 4B). This suggested lower GRF6 stability in the presence of MPK11. The ubiquitin-proteasome system (UPS) is a major component of the cell machinery engaged in the control of protein abundance, and plays an important role in the regulation of multiple biological processes [16]. UPS target proteins are ubiquitinated by E3 ubiquitin ligases, recognized by the 26S proteasome complex and degraded. Therefore, we analyzed GRF6 ubiquitination following transient expression in *N. benthamiana* and immunoprecipitation with anti-HA antibodies. Western blot analysis using anti-ubiquitin monoclonal antibodies revealed that a fraction of immunoprecipitated GRF6 was ubiquitinated (Fig. 4C, left panel), accumulating predominantly as a likely tetraubiquitinated form according to its estimated molecular mass. To confirm the identity of this ubiquitinated protein as GRF6, a Western blot was immunodecorated with anti-HA antibodies, revealing detection of the same protein along with the fast migrating and more abundant unmodified GRF6 protein (Fig. 4C, right panel). Interestingly, abundance of the ubiquitinated fraction increased when GRF6 was coexpressed with MPK11, suggesting a higher ubiquitination rate upon MPK11-mediated GRF6 phosphorylation. This correlated with the increased GRF6 instability we observed in the presence of MPK11, as shown in Fig. 4B. Reinforcing the functional interrelationship existing among these three proteins, DBP1 prevented ubiquitination of GRF6. When DBP1 and MPK11 were simultaneously expressed along with GRF6 in *N. benthamiana* leaves, the presence of DBP1 restricted MPK11-promoted GRF6 ubiquitination (Fig. 4C). This was likely due to the inhibitory effect of DBP1 on MPK11 activity which, as shown above, also prevented GRF6 phosphorylation mediated by MPK11 (Fig. 4A).

### Candidate DBP1 Targets had Different Roles in PPV Infection

**Figure 5:**
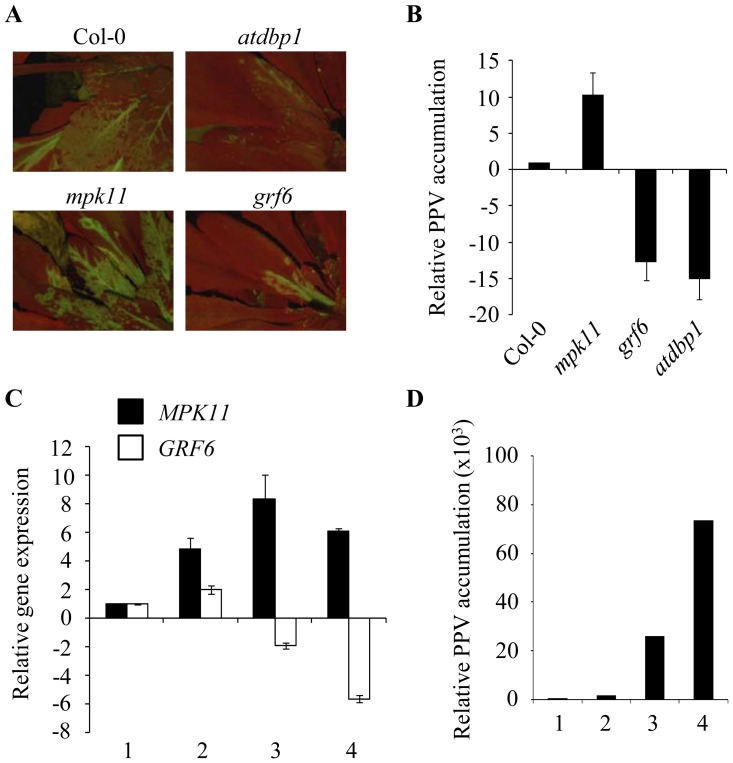
Putative DBP1 targets played a role in PPV infection, positively or negatively affecting PPV multiplication. A, Progression of PPV infection in loss-of-function mutants. Plants of the indicated genotypes were inoculated with PPV-GFP and infection extent was analyzed by fluorescence microscopy to monitor GFP distribution. B, PPV accumulation in loss-of-function mutants. Viral accumulation was measured by RT-qPCR using primers specifically amplifying the viral coat protein gene. Data were normalized using *ACT2/8* as a reference gene. Relative accumulation with respect to Col-0 is shown as the mean of three independent experiments ± SD. C, Gene expression analysis during the course of PPV-GFP infection in Col-0 plants. Inoculated leaf tissue showing productive infection was pooled into four different categories according to the degree of viral colonization determined under the fluorescence microscope. Leaf material with no visible (1), low (2), medium (3), and high (4) viral accumulation was harvested, and gene expression was analyzed by RT-qPCR using *ACT2/8* as a reference gene. Expression data were normalized to those obtained for similar samples collected from uninfected plants. The mean of three independent experiments ± SD is shown. D, PPV accumulation in the selected infection categories. Viral accumulation was quantified by RT-qPCR as above, and referred to the value in (1). Although no visible signal was observed in these leaves when assessed microscopically, there was an incipient infection detectable by RT-qPCR.

DBP1 functions as a host susceptibility factor during PPV infection [3], whereas DIP2, a small polypeptide functionally modulating DBP1, contributes to resistance [4]. The implication of the selected proteins in the Arabidopsis-PPV interaction was examined by selecting homozygous mutant plants bearing a T-DNA insertion in the relevant gene that markedly reduces gene expression (Fig. S1A). Plants of these lines were inoculated with a PPV-GFP viral strain, and infection progression was monitored by fluorescence microscopy and by RT-qPCR using specific primers for the viral coat protein gene. Interestingly, *mpk11* showed increased susceptibility to PPV, whereas *grf6* exhibited enhanced resistance (Fig. 5A, B). Complementation of the analyzed mutants with genomic fragments bearing the wild-type gene sequence showed that the observed phenotypes were indeed due to loss of function of the relevant genes (Figure S1C).

As both genes appeared to be implicated in the Arabidopsis-PPV interaction, we analyzed their expression dynamics relative to viral accumulation in Col-0. PPV-inoculated plants were inspected under the fluorescence microscope and infected leaves were grouped into four different categories based on the degree of viral colonization. Leaf material exhibiting no (1), low (2), medium (3), and high (4) viral accumulation were harvested, and gene expression was analyzed by RT-qPCR, referred to *ACT2/8* and normalized using similar samples from uninfected plants (Fig. 5C). *MPK11* transcript accumulation increased during infection, while *GRF6* expression was repressed following MPK11 induction. Fig. 5D shows increasing viral accumulation through the selected categories as quantified by RT-qPCR.

**Figure 6:**
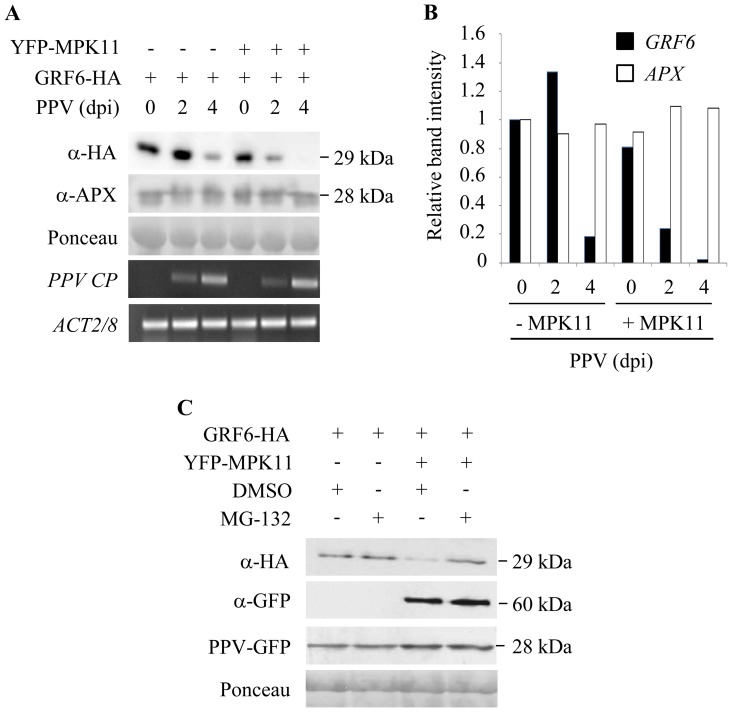
MPK11 promotes increased GRF6 protein turn-over during PPV infection. A, Analysis of GRF6 accumulation in infection by PPV. GRF6-HA was transiently expressed in *N. benthamiana* leaves alone or together with MPK11 as indicated. Protein accumulation was analyzed 3 days post-infiltration in leaves that were not inoculated with PPV (0 dpi), leaves that were inoculated with PPV one day after GRF6-HA agroinfiltration (2 dpi; in these leaves, PPV infection is at day 2 when GRF6 protein accumulation was analyzed), and leaves that were inoculated with PPV one day before GRF6-HA agroinfiltration (4 dpi; in these leaves, PPV infection is at day 4 when GRF6-HA accumulation was recorded). As an internal control, accumulation of ascorbate peroxidase (APX) is shown. Ponceau-S staining of membrane indicates equal protein loading. PPV and *ACTIN2/8* RNA accumulation was determined by RT-PCR. B, Quantification of GRF6 and APX bands in Western blot using ImageJ software. Values were referred to those in the absence of PPV and MPK11. The experiment was repeated three times with similar results. C, Proteasome mediates GRF6 degradation promoted by MPK11. GRF6-HA and YFP-MPK were transiently expressed in *N. benthamiana* leaves 4 days after inoculation of the same leaves with PPV. Two days later, leaves were infiltrated either with the proteasome inhibitor MG-132 or with the same concentration of the solvent DMSO, and after 24 h, leaf material was collected. Protein accumulation was analyzed by Western blot using specific antibodies as indicated.

To demonstrate the biological relevance of these findings, we analysed GRF6 accumulation during the course of PPV infection in *N. benthamiana*. Protein level was measured after 3 d of expression at different time points of infection (Fig. 6A, B). As described above, GRF6 accumulation was reproducibly diminished when coexpressed with MPK11. Here, the magnitude of the decrease observed in non-inoculated tissue (0 dpi) is lower than that shown earlier. This is not surprising, since de-stabilization of GRF6 mediated by MPK11 depends on the expression level of MPK11 and also on the degree of activation of the kinase, which relies on endogenous MAPKKs. A significant further reduction in GRF6 protein accumulation was observed 4 d post-inoculation with PPV. This reduction was specific and not observed for cytosolic ascorbate peroxidase (APX), here used as a negative control, neither by Ponceau-S staining of the protein extracts. GRF6 protein decline was markedly accelerated in the presence of MPK11, corroborating the increased GRF6 turn-over caused by MPK11. To ascertain the implication of the proteasome in this process, GRF6 and MPK11 were transiently expressed in leaves of *N. benthamiana* which had been previously inoculated with PPV in order to promote activation of MPK11 and GRF6 degradation. As shown in Fig. 6C, treatment with the proteasome inhibitor MG-132 24 h prior to collecting the tissue reverted the reduction in GRF6 stability observed when coexpressed with MPK11, indicating that the proteasome is mediating GRF6 degradation promoted by MPK11. In contrast, in the absence of MPK11, addition of MG-132 did not have any significant effect on GRF6 protein accumulation, as expected if phosphorylation of GRF6 by MPK11 is the molecular event that targets GRF6 for degradation via the proteasome.

## Discussion

DBPs are plant-specific DNA-binding protein phosphatases [1], [2], and have been found to play a role in plant-virus interactions [1], [3], [4]. However, little is known about the molecular mechanisms in which DBP factors are involved. Therefore, we searched for putative DBP1 targets by analysing phosphoproteome changes resulting from *DBP1* loss-of-function. From the identified proteins we selected 14-3-3λ (GRF6) and MPK11 for further analyses.

GRF6 is λ isoform within the 14-3-3 family, highly conserved proteins involved in regulating multiple biological processes and signalling pathways in eukaryotes. 14-3-3 proteins usually bind phosphopeptide motifs and modify function of their targets at different levels [17]. GRF6 was previously shown to interact with DBP1 using the yeast two-hybrid system [6], an interaction conserved in tobacco and Arabidopsis. MPK11 is one of the about 20 MAPKs present in Arabidopsis. Together with MAPKKs and MAP3Ks, MAPKs form signalling cascades mediating developmental processes and responses to both biotic and abiotic stresses in all eukaryotic organisms. Identification of substrates and factors regulating kinase activity is essential for a better understanding of cell responses to developmental, hormone and environmental signals. MAPKs are activated by phosphorylation, whereas dephosphorylation leads to inactivation. In yeast and mammals, tyrosine and dual specificity phosphatases are considered the major MAPK phosphatases [18]. In plants, serine/threonine protein phosphatases 2C have also been shown to dephosphorylate and modulate MAPK activity [19]–[23]. When activated, MAPKs phosphorylate a variety of substrates, thereby modulating their function. Here we show that DBP1 negatively regulated MPK11 activity and that GRF6 seems to be a MPK11 substrate. In Arabidopsis, only a few MAPK targets have been identified *in vivo* so far. Popescu et al. (2009) undertook an extensive search for substrates of 10 MAPKs using protein microarrays, but MPK11 was not included in the study [24]. We demonstrated that GRF6 interacts with MPK11, and that MPK11 promotes phosphorylation of GRF6 *in vivo*. Moreover, DBP1 prevented MPK11-mediated GRF6 phosphorylation. Interestingly, GRF6 accumulated at lower levels when coexpressed with MPK11, suggesting a phosphorylation-dependent regulation of GRF6 protein abundance. Indeed, GRF6 was ubiquitinated *in vivo*, and ubiquitination was enhanced in the presence of MPK11, leading to higher degradation rates. In contrast, the presence of DBP1 protected GRF6 from the increased ubiquitination promoted by MPK11. This is likely a consequence of the inhibition of MPK11 activity exerted by DBP1. Moreover, GRF6 degradation was attenuated in the presence of the proteasome inhibitor MG-132, indicating that GRF6 is degraded via the proteasome. These results provide compelling evidence for a regulatory protein network nucleated by DBP1 which modulates stability of GRF6, with GRF6 in turn mediating nucleo-cytoplasmic shuttling of DBP1. A ubiquitin ligase was recently reported to target 14-3-3 proteins in a stimulus-specific manner in the cellular C/N balance response [25]. Therefore, other 14-3-3 proteins are subjected to a similar regulation through induced degradation via UPS.

MPK11 promoted enhanced GRF6 degradation, negatively regulating GRF6 function. BiFC analysis provides a cellular basis to better interpret these biochemical data. As DBP1, GRF6 also localized both to the nucleus and the cytosol (Fig. 1), and, in turn, interaction between these two factors, as detected by BiFC, was also observed in the two cellular compartments (Fig. 2A). Accumulation of GRF6-DBP1 complex in the cytosol is congruent with DBP1 relocalization from the nucleus to the cytosol, as promoted by 14-3-3 G in tobacco [6]. In contrast, the interaction between GRF6 and MPK11 was detected predominantly in the nucleus. MPK11 could be preventing GRF6-DBP1 interaction in the nucleus by promoting GRF6 degradation. Phosphorylation is well known to influence ubiquitination either positively or negatively [26]. Thus, the activity of E3 ubiquitin ligases may be regulated by phosphorylation. Alternatively, phosphorylation of the target protein can enable or prevent E3 ubiquitin ligase recognition, or regulate E3 ubiquitin ligase access to its target by modifying its subcellular localization. In the case of GRF6, both mechanisms are possible. Phosphorylation by MPK11 might create a binding site for E3 ubiquitin ligase, or could recruit GRF6 to the nucleus, where the E3 ubiquitin ligase might be located. Although the MPK11-GRF6 complex accumulates in the nucleus, we cannot exclude that the interaction takes place in the cytosol, and promotes rapid nuclear targeting of GRF6 for ubiquitination, thereby preventing interaction with DBP1.

Little is known about the biology of DBP factors. DBP1 was recently reported to act as a potyvirus susceptibility factor, since the absence of *DBP1* function hindered infection by the potyviruses *Plum pox virus* (PPV) and *Turnip mosaic virus* (TuMV) [3]. Complex sets of host-pathogen interactions determine the outcome of plant virus infection. These interactions, the underlying mechanisms and the involved signalling pathways are largely unknown. We showed that both *MPK11* and *GRF6* also play a role in the Arabidopsis-PPV interaction, since the respective loss-of-function mutants exhibited an altered response to PPV infection. MPK11 emerges as a signalling factor likely mediating the defense response to PPV infection, since loss of function leads to increased susceptibility. Three weeks after inoculation, PPV accumulation was approximately 10-fold higher in the *mpk11* mutant than in Col-0 plants, as determined by RT-qPCR (Fig. 5A). The fact that *MPK11* expression was moderately and transiently induced during infection supports this hypothesis (Fig. 5C). This assigns a biological role to MPK11, and sheds light on the signalling processes triggered in response to PPV infection. Therefore, MPK11 shows promise for engineering improved resistance against PPV. In contrast, *GRF6*-deficient plants displayed enhanced resistance to PPV infection, a phenotype similar to that of *dbp1* mutants, suggesting that DBP1 and GRF6 likely cooperate to confer susceptibility, whereas MPK11 would be antagonizing this effect. Since MPK11 promotes GRF6 degradation, to test whether this regulation plays a role in the Arabidopsis-PPV interaction, we analyzed GRF6 accumulation during the course of PPV infection in *N. benthamiana* leaves (Fig. 6A). In PPV-inoculated leaves, GRF6 protein level was significantly reduced as the virus accumulated in the locally infected tissue. However, when co-expressed with MPK11, GRF6 turnover was specifically accelerated. This further suggests that MPK11 would be involved in a plant local defense response aimed at reducing GRF6 protein level in early stages of PPV infection increasing its proteasome-mediated degradation.

Therefore, our results have a significant impact not only in the biology of DBP factors, but also contribute to a better knowledge of MAPK signalling pathways in Arabidopsis through the identification of GRF6 as a likely MPK11 substrate and of DBP1 as a protein phosphatase regulating MPK11 activity, and unveils the implication of this protein module in the response to PPV infection in Arabidopsis.

## Supporting Information

Figure S1 **T-DNA insertion mutants used in this study.** A, RT-qPCR analysis of gene expression. Data were normalized using *ACT2/8* as a reference gene and are expressed relative to the respective expression level in wild-type Col-0 plants. B, Images of 4 week-old representative plants of each mutant line compared to the wild-type Col-0 ecotype. No visible alteration was observed in plant morphology and architecture at any developmental stage, except for *mpk4* mutant which showed severe developmental defects. C, *mpk11* and *grf6* mutant lines were transformed with genomic fragments encompassing the corresponding wild-type structural genes and more than 1500 bp of promoter sequence. Homozygous plants bearing single insertions were inoculated with PPV and progression of infection was analyzed by RT-qPCR using primers for the viral coat protein gene. Expression values were normalized using *ACT2/8* and referred to Col-0.
(TIF)

Figure S2 **Specificity of the antibodies used in immunoprecipitation assays.** The indicated protein combinations were transiently expressed in *N. benthamiana* leaves by agroinfiltration, and immunoprecipitated with the antibodies indicated on top. The corresponding immunoprecipitated fractions were analyzed by Western blot using the antibodies referred on the left.
(TIF)

Methods S1 (DOCX)

Table S1 (XLS)
