# Supplementary figures and images for: Arabidopsis Protein Phosphatase DBP1 Nucleates a Protein Network with a Role in Regulating Plant Defense
Source: PLoS One. 2014 Mar 4;9(3):e90734. doi: 10.1371/journal.pone.0090734 (PMC3942490; doi:10.1371/journal.pone.0090734)

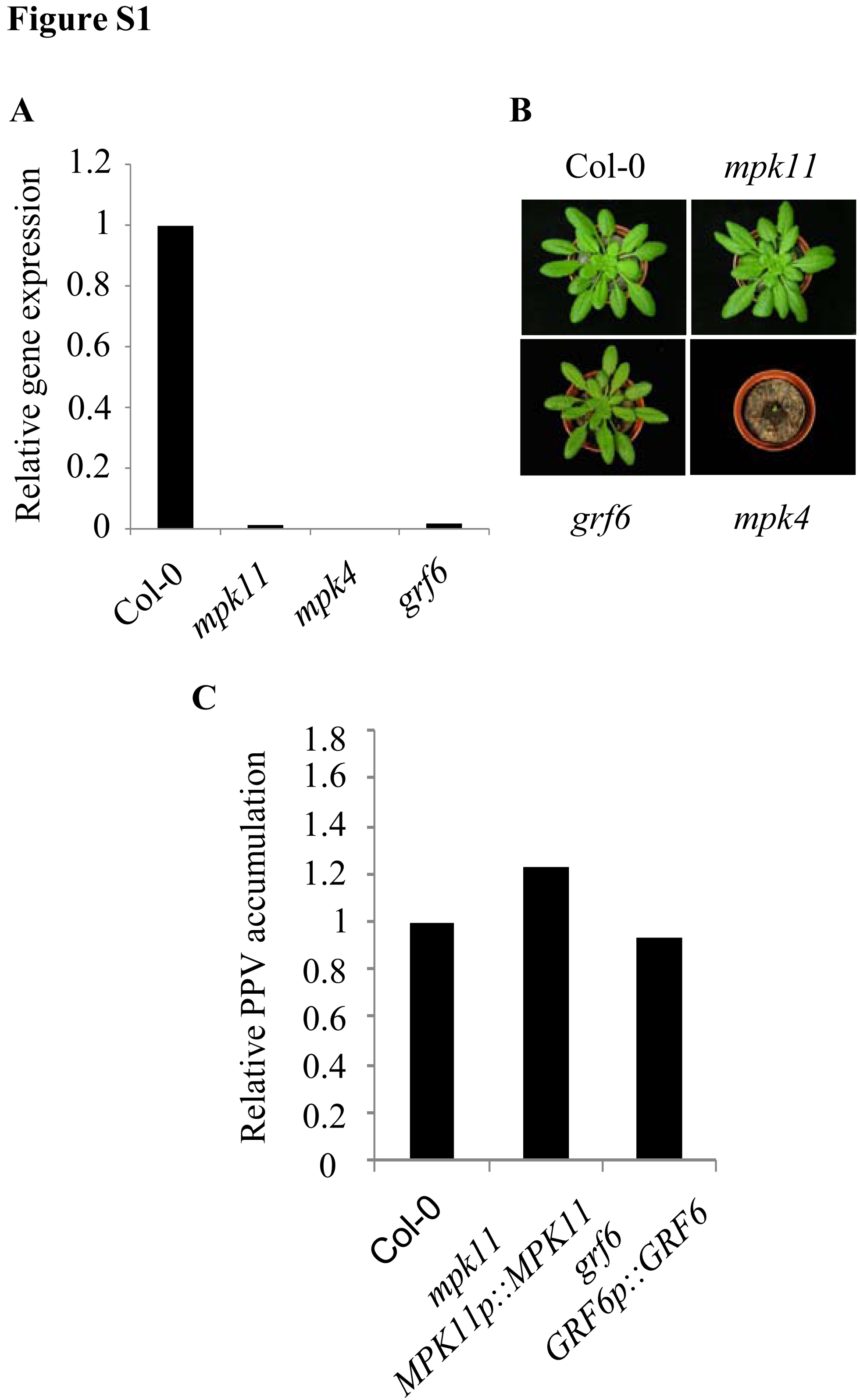

Supplement: Figure S1 — T-DNA insertion mutants used in this study. A, RT-qPCR analysis of gene expression. Data were normalized using ACT2/8 as a reference gene and are expressed relative to the respective expression level in wild-type Col-0 plants. B, Images of 4 week-old representative plants of each mutant line compared to the wild-type Col-0 ecotype. No visible alteration was observed in plant morphology and architecture at any developmental stage, except for mpk4 mutant which showed severe developmental defects. C, mpk11 and grf6 mutant lines were transformed with genomic fragments encompassing the corresponding wild-type structural genes and more than 1500 bp of promoter sequence. Homozygous plants bearing single insertions were inoculated with PPV and progression of infection was analyzed by RT-qPCR using primers for the viral coat protein gene. Expression values were normalized using ACT2/8 and referred to Col-0. (TIF) [file pone.0090734.s001.tif]

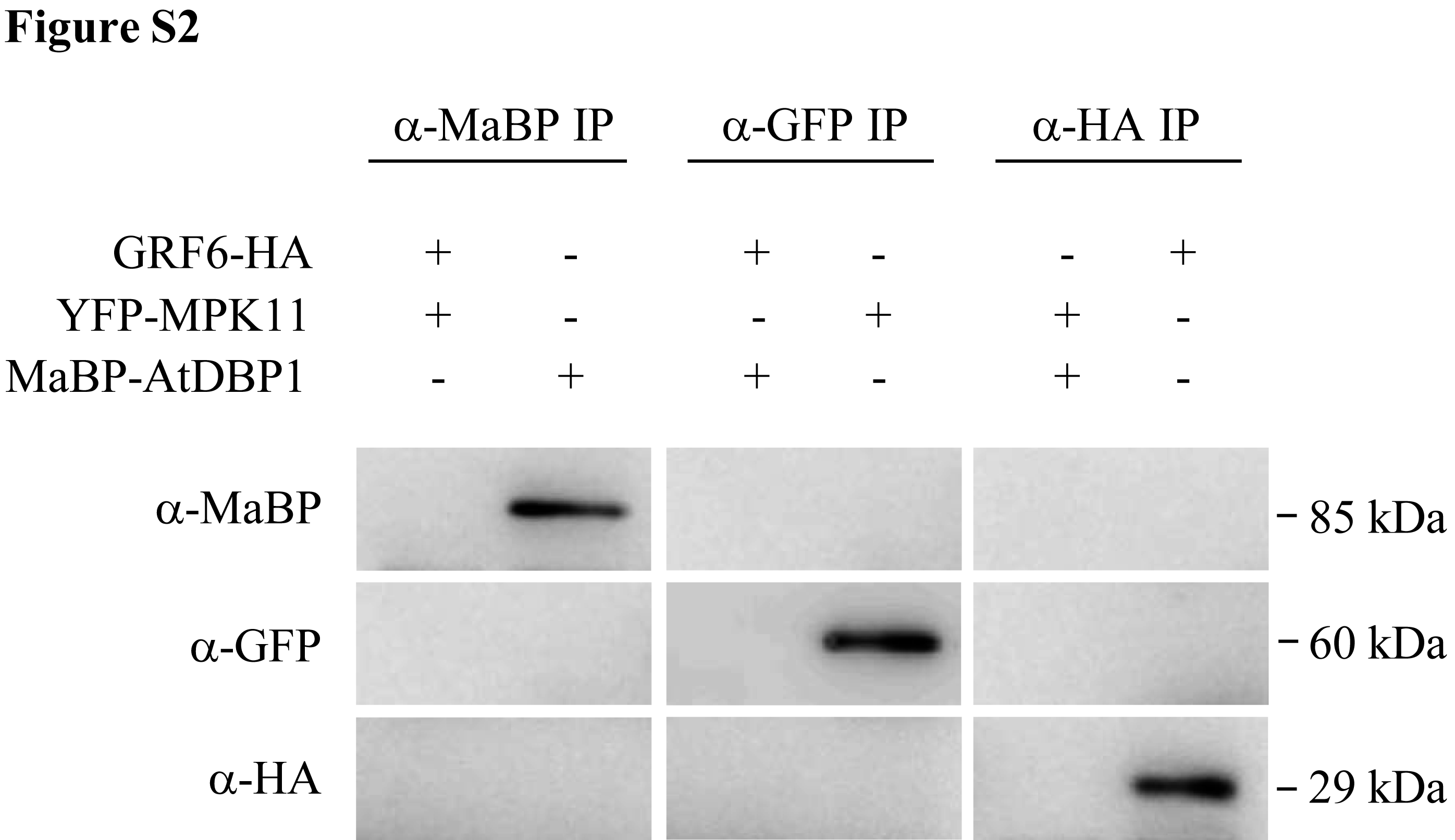

Supplement: Figure S2 — Specificity of the antibodies used in immunoprecipitation assays. The indicated protein combinations were transiently expressed in N. benthamiana leaves by agroinfiltration, and immunoprecipitated with the antibodies indicated on top. The corresponding immunoprecipitated fractions were analyzed by Western blot using the antibodies referred on the left. (TIF) [file pone.0090734.s002.tif]
