# Supplementary material for: Arabidopsis Protein Phosphatase DBP1 Nucleates a Protein Network with a Role in Regulating Plant Defense
Source: PLoS One. 2014 Mar 4;9(3):e90734. doi: 10.1371/journal.pone.0090734 (PMC3942490; doi:10.1371/journal.pone.0090734)
Supplement: Methods S1 — (DOCX) [file pone.0090734.s003.docx]

**METHODS S1**

**Phosphoprotein enrichment and LC-MS analysis**

*Protein Extraction and Phosphoprotein Enrichment*

*Arabidopsis* leaf material was ground in liquid nitrogen and mixed with extraction buffer. The suspension was centrifuged and the supernatant was used for RuBisCO removal by ammonium sulfate. The precipitated proteins were air-dried and solubilized in an adequate buffer volume for adapted metal-oxide affinity chromatography (MOAC) [1]. Eluted phosphoproteins were centrifuged and subsequently concentrated by using a filter device. Finally, proteins were precipitated with 2D-CleanUp Kit (GE Healthcare) according to manufacturer’s manual and solubilized in lysis buffer (8 M urea, 50 mM Tris-HCl, pH 8.5).

*In-solution Digestion with Trypsin*

Phosphoproteins were reduced and alkylated. Further, the proteins were digested with trypsin (Promega) overnight at 37 °C. Tryptic peptides were desalted and reconstituted in a solution containing 5% ACN (v/v) and 0.1% TFA (v/v).

*Liquid Chromatography - Tandem Mass Spectrometry (LC-MS/MS) - LTQ Orbitrap Velos System*

Tryptic digests of enriched phosphoproteins were analyzed with a LC-MS system consisting of a nano-LC (Easy-nLC II, Thermo Fisher Scientific, Bremen, Germany) coupled to an hybrid-FT-mass spectrometer (LTQ Orbitrap Velos, Thermo Fisher Scientific, Bremen, Germany). Peptide separations were performed on C_18_ column at a flow rate of 300 nL/min and a linear gradient from 2 to 40% B in 150 min (A: 0.1% formic acid in water, B: 0.1% formic acid in acetonitrile). Auto-MS/MS measurements were performed by exclusion of singly charged ions. Precursor mass scan was performed from 400 to 1850 m/z in the Orbitrap with resolution 30,000 and 20 precursors were selected per full scan for subsequent CID fragmentation in the LIT.

*Data Analysis and Evaluation*

MS raw data were processed (DataAnalysis 4.0 and Proteome Discoverer 1.3) and subsequently searched with an in-house Mascot server (Version 2.2/3, Matrix Science, London, UK) against an *A. thaliana* protein database based on TAIR 10. For further data evaluation the Scaffold software (Version 3.3, Proteome Software Inc*.*, Portland, OR, USA) was used.

**Constructs for mutant complementation**

For *mpk11* and *grf6* mutant complementation, genomic fragments encompassing at least 1500 bp of promoter sequence along with the structural genes and 5' and 3' untranslated regions were cloned into the vector pMDC99 using Gateway technology [2].

**REFERENCES**

1. Wolschin F, Wienkoop S, Weckwerth W (2005) Enrichment of phosphorylated proteins and peptides from complex mixtures using metal oxide/hydroxide affinity chromatography (MOAC). Proteomics 5: 4389-4397.

2. Curtis M, and Grossniklaus U (2003) A Gateway cloning vector set for high-throughput functional analysis of genes in plants. [Plant Physiol 133: 462-469](http://botserv1.uzh.ch/home/grossnik/curtisvector/gateway.pdf).
